# The Clinical Characteristics and Management of Dental-Related Foreign Body Accidents: A Retrospective Observational Study

**DOI:** 10.3390/healthcare14193164

**Published:** 2026-09-24

**Authors:** Shintaro Sukegawa, Miya Okanishi, Fumi Nakai, Yasuhiro Nakai, Masato Saika, Minoru Miyake

**Affiliations:** 1 Department of Oral and Maxillofacial Surgery, Kagawa University Faculty of Medicine, 1750-1 Ikenobe, Miki-cho, Kita-gun, Kagawa 761-0793, Japan; 2 Department of Oral Pathology and Medicine, Graduate School of Medicine, Dentistry and Pharmaceutical Sciences, Okayama University, Okayama 700-8558, Japan; 3 Division of Dental Anesthesiology and Pain Management, Graduate School of Dentistry, Kyushu Dental University, 2-6-1 Manazuru, Kokurakita-ku, Kitakyushu, Fukuoka 803-8580, Japan; 4 Department of Anesthesiology, Kurume University School of Medicine, 67 Asahi-machi, Kurume, Fukuoka 830-0011, Japan

**Keywords:** foreign bodies, dentistry, Endoscopy, gastrointestinal, patient safety, foreign body aspiration

## Abstract

**Background**: Dental-related foreign body accidents are uncommon but potentially serious iatrogenic complications. Although numerous case reports have been published, comprehensive clinical studies describing their clinical characteristics, management, and outcomes remain limited. This study investigated the clinical characteristics, management, and outcomes of dental-related foreign body accidents. **Patients and Methods**: We conducted a retrospective observational study of patients with dental-related foreign body accidents treated at a single tertiary referral center between April 2016 and March 2026. Patient demographics, foreign body characteristics, imaging findings, management, and clinical outcomes were retrospectively reviewed. **Results**: Twenty-six patients were included, comprising 25 cases of foreign body ingestion and one case of aspiration. Restorative materials were the most common foreign bodies (57.7%), followed by dental instruments (15.4%) and prosthetic appliances (11.5%). Endoscopic removal was performed in 12 patients (46.2%), whereas the patient with aspiration underwent bronchoscopic removal. All endoscopic procedures were successfully performed on the day of presentation. No foreign body-related or management-related complications were observed during follow-up. Owing to the retrospective single-center design and limited sample size, these findings should be interpreted with caution. **Conclusions**: This retrospective observational study provides real-world clinical data on the characteristics, management, and outcomes of dental-related foreign body accidents. Restorative materials were the most common foreign bodies, and approximately half of the patients required endoscopic removal.

## 1. Introduction

The ingestion of a foreign body is a relatively common clinical problem encountered in emergency medicine and gastrointestinal practice [1,2]. Most ingested foreign bodies pass spontaneously through the gastrointestinal tract without complications, but approximately 10–20% of such cases require endoscopic intervention, and <1% require surgical treatment [2]. A delayed diagnosis or inappropriate management may result in serious complications such as gastrointestinal perforation, obstruction, or bleeding, particularly in cases involving sharp or elongated objects [3]. The clinical guidelines recently published by the European Society of Gastrointestinal Endoscopy (ESGE) emphasize the importance of a prompt imaging evaluation and appropriate endoscopic management according to the size, shape, and location of the ingested foreign body [4,5].

Dental-related foreign body ingestions have unique clinical characteristics compared to general gastrointestinal foreign body ingestions. The foreign bodies associated with dental treatment are often small, sharp, and metallic, and they include dental burs, crowns, inlays, implant components, orthodontic appliances, and prosthetic materials [6,7]. In addition, many cases are considered iatrogenic events occurring during dental procedures, particularly under conditions of limited visibility and the use of small instruments in the oral cavity [8]. Although most dental foreign bodies pass spontaneously, some cases require urgent endoscopic intervention because of the risk of mucosal injury or gastrointestinal perforation caused by a sharp and irregularly shaped object [4]. The prompt recognition, appropriate imaging evaluation, and multidisciplinary collaboration between dentists and gastroenterologists are thus important for the safe management of these patients.

Although previous retrospective studies have described the incidence, characteristics, and management of dental-related foreign body accidents [6,7,8,9], studies comprehensively evaluating patient characteristics, foreign body characteristics, multidisciplinary management, and clinical outcomes within a single clinical cohort remain limited. Therefore, we conducted this retrospective observational study to investigate the clinical characteristics, management, and clinical outcomes of patients with dental-related foreign body accidents.

## 2. Patients and Methods

### 2.1. Study Design and Patients

We conducted a retrospective single-center observational study at the Department of Oral and Maxillofacial Surgery, Kagawa University Hospital. Patients diagnosed with dental-related foreign body ingestion or aspiration between April 2016 and March 2026 were included. Dental-related foreign body accidents were defined as ingestion or aspiration associated with dental treatment, dental materials, prostheses, or oral appliances. Cases involving non-dental foreign bodies (e.g., food-related foreign bodies, coins, and medication packaging) were excluded. Clinical records, radiographic findings, treatment details, and outcomes were obtained from the patients’ electronic medical records. All included patients had complete medical records, radiographic findings, and clinical outcome data. No patients were excluded because of incomplete records, repeat admissions, insufficient follow-up, or missing essential clinical information. During the study period, 29 patients with foreign body ingestion or aspiration were initially identified. Three patients (one button battery and two coin ingestions) were excluded because the foreign bodies were unrelated to dental treatment, leaving 26 patients for the final analysis.

### 2.2. Ethics Statement

This study was approved by the institutional review board of Kagawa University Hospital (approval no. 2026-067). In light of the study’s retrospective nature, the requirement for patients’ informed consent was waived.

### 2.3. Data Collection

The following variables were evaluated: age, sex, body mass index (BMI), type and size of the foreign body, patient symptoms at presentation, imaging results, endoscopic intervention, follow-up abdominal radiography, consultation with gastroenterology, and clinical outcomes. Clinical data were retrospectively collected by one investigator using a standardized data extraction form. Symptoms at presentation were defined as any patient-reported or clinically documented symptoms recorded in the medical records at the initial evaluation, including throat discomfort, dysphagia, chest discomfort, abdominal pain, cough, or no symptoms. Clinical outcomes were defined as successful endoscopic or bronchoscopic removal; spontaneous passage or expectoration confirmed during follow-up, hospitalization, and discharge; and the occurrence of procedure-related or foreign body-related complications. Complications were defined as documented adverse events related to the foreign body itself or its clinical management during follow-up.

### 2.4. Management Strategy

The patients had been evaluated using radiographic imaging (including plain radiography, panoramic radiography, and computed tomography) according to the clinical situation. Following the initial assessment by oral and maxillofacial surgeons, all of the patients’ cases had been discussed between the oral and maxillofacial surgeons and gastroenterologists. Information regarding the type, shape, and location of the foreign body was shared between the oral and maxillofacial surgeons and gastroenterologists. The decision regarding endoscopic intervention was based on the type, size, shape, and anatomical location of the foreign body, together with the patient’s clinical condition and radiological findings, with reference to the ESGE guideline. Although the ESGE clinical guideline was used as a reference in clinical practice, the final management strategy was determined at the discretion of the attending gastroenterologist.

### 2.5. Statistical Analyses

Continuous variables are presented as mean ± standard deviation (SD), and categorical variables are presented as frequencies and percentages. Because the primary objective of this study was descriptive and the sample size was limited, no comparative or multivariable analyses were performed. The statistical analyses were performed using JMP version 19.0 (SAS Institute Inc., Cary, NC, USA).

## 3. Results

### 3.1. Patient Characteristics

The cases of a total of 26 patients with dental-related foreign body accidents were analyzed. Among them, 25 patients (96.2%) ingested a foreign body, and the remaining patient (3.8%) aspirated a foreign body requiring bronchoscopic removal. The patients’ mean age was 58.3  ±  26.6 years. Sixteen patients (61.5%) were male, and the other 10 patients (38.5%) were female. The mean BMI was 22.0  ±  4.0 kg/m^2^.

Three patients (11.5%) were <18 years old, 12 (46.2%) were aged 18–64 years, and 11 (42.3%) were ≥65 years. Nineteen patients (73.1%) experienced foreign body accidents during dental treatment, whereas seven patients (26.9%) experienced them outside dental treatment. Two patients (7.7%) were transported to the hospital by ambulance, and 12 (46.2%) were referred from dental clinics. The patient characteristics are summarized in Table 1.

### 3.2. Foreign Body Characteristics

The characteristics of the foreign bodies are summarized in Table 2. The clinical characteristics, management, and outcomes of the individual patients are presented in Supplementary Table S1. Restorative materials constituted the largest category of foreign bodies, accounting for 15 cases (57.7%), followed by dental instruments (4 cases, 15.4%), prosthetic appliances (3 cases, 11.5%), tooth fragments (2 cases, 7.7%), orthodontic appliances (1 case, 3.8%), and metal fragments (1 case, 3.8%). The mean long-axis diameter of the foreign bodies was 16.8 ± 9.7 mm, and the mean short-axis diameter was 7.3 ± 3.8 mm. Most foreign bodies were radiopaque and could be identified by conventional radiographic examinations.

### 3.3. Clinical Management and Outcomes

The clinical management and outcomes are summarized in Table 3. Plain radiographs were available for all patients, either at presentation or from referring institutions. Panoramic radiography and computed tomography were performed in five (19.2%) and ten (38.5%) patients, respectively. Following the initial assessment by oral and maxillofacial surgeons, all cases were discussed with gastroenterologists, including telephone consultation. Subsequently, 17 patients (65.4%) underwent evaluation by the gastroenterology service. Endoscopic removal was performed in 12 patients (46.2%), whereas one patient (3.8%) with foreign body aspiration underwent bronchoscopic removal. The remaining 13 patients were managed conservatively and followed in the outpatient clinic. Follow-up abdominal radiography was performed in 11 patients (42.3%) to confirm spontaneous passage of the foreign body when clinically indicated. No complications related to the foreign body or its management were observed during follow-up. The individual clinical characteristics, management, and outcomes of all patients are summarized in Supplementary Table S1. The overall clinical management pathway is illustrated in Figure 1.

## 4. Discussion

This retrospective study described the clinical characteristics, management, and outcomes of dental-related foreign body accidents treated at a tertiary referral center. Restorative materials were the most common foreign bodies, 12 of the 26 patients underwent endoscopic removal, and no complications were observed during follow-up. These findings provide clinically relevant real-world data regarding the management of this uncommon dental emergency.

Restorative materials represented the largest proportion of foreign bodies in this patient cohort, accounting for more than half of the 26 cases. This finding is consistent with reports that crowns, fixed dental prostheses, and other restorative materials are the most frequently ingested dental objects during dental treatment [6,10]. In particular, Huh et al. reported that fixed dental prostheses and implant components were the most commonly ingested objects in a university dental hospital, suggesting that restorative and prosthodontic procedures may be associated with an increased risk of dental foreign body accidents [9]. Although dental instruments and orthodontic appliances were less common in our present cohort, these objects warrant particular attention because their sharp or irregular configurations increase the risk of gastrointestinal injury and frequently necessitate urgent endoscopic retrieval [4]. Collectively, these findings indicate that restorative and prosthodontic procedures warrant particular attention because they accounted for the majority of dental-related foreign body accidents in the present cohort.

Nearly half of the patients in our cohort underwent endoscopic removal, and all of the endoscopic procedures were successfully performed on the day of presentation without procedure-related complications. Although the indication for endoscopic intervention was ultimately determined by the attending gastroenterologist, oral and maxillofacial surgeons played an essential role in the initial assessment by identifying the foreign body and communicating its characteristics, including its type, shape, and anatomical location. In the present cohort, patient management was undertaken through close collaboration between oral and maxillofacial surgeons and gastroenterologists.

The current ESGE guidelines recommend urgent endoscopic removal for sharp-pointed or otherwise high-risk foreign bodies because of the potential for gastrointestinal injury [2,4]. However, the practical management of dental-related foreign body accidents requires multidisciplinary collaboration, as the characteristics of the ingested object and the clinical circumstances vary considerably among patients [9].

The prevention of dental-related foreign body accidents is equally important. Preventive measures such as the routine use of rubber dams, floss ligatures, and gauze throat screens have been recommended in previous studies and clinical guidelines [10]. Although the effectiveness of these measures was not evaluated in the present study, their routine use during restorative and prosthodontic procedures is supported by the existing literature.

This study has several limitations. First, it was a retrospective study conducted at a single tertiary referral center, which may limit the generalizability of the findings. The tertiary referral-center setting may also have introduced referral and selection bias because patients with more complex or higher-risk foreign body accidents may have been preferentially referred. Second, the sample size was relatively small because dental-related foreign body accidents are uncommon. Third, treatment decisions, including the indication for endoscopic removal, were made according to routine clinical practice rather than a predefined standardized protocol and may therefore have varied among patients. Fourth, follow-up imaging was not routinely performed in all conservatively managed patients, and the completeness and duration of follow-up varied according to clinical judgment. Consequently, delayed complications or spontaneous passage may not have been uniformly assessed in all patients. Fifth, because of the descriptive nature of the study and the limited sample size, no comparative or adjusted analyses were performed. Therefore, potential confounding factors, including patient age, comorbidities, and the type, size, and anatomical location of the foreign body, could not be controlled. The observed management outcomes should therefore not be interpreted as evidence of causal relationships or the comparative effectiveness of specific management strategies. Nevertheless, this study provides detailed real-world descriptive data regarding the clinical characteristics and management of dental-related foreign body accidents. Larger multicenter studies using standardized definitions, data-collection procedures, management protocols, and follow-up methods are needed to identify factors associated with clinical intervention and patient outcomes.

## 5. Conclusions

This retrospective observational study describes the clinical characteristics, management, and outcomes of dental-related foreign body accidents encountered at a tertiary referral center. Restorative materials were the most common foreign bodies, and approximately half of the patients required endoscopic removal. These findings provide real-world clinical data that may contribute to a better understanding of dental-related foreign body accidents and may assist clinicians in their clinical assessment and management.

## Figures and Tables

**Figure 1:**
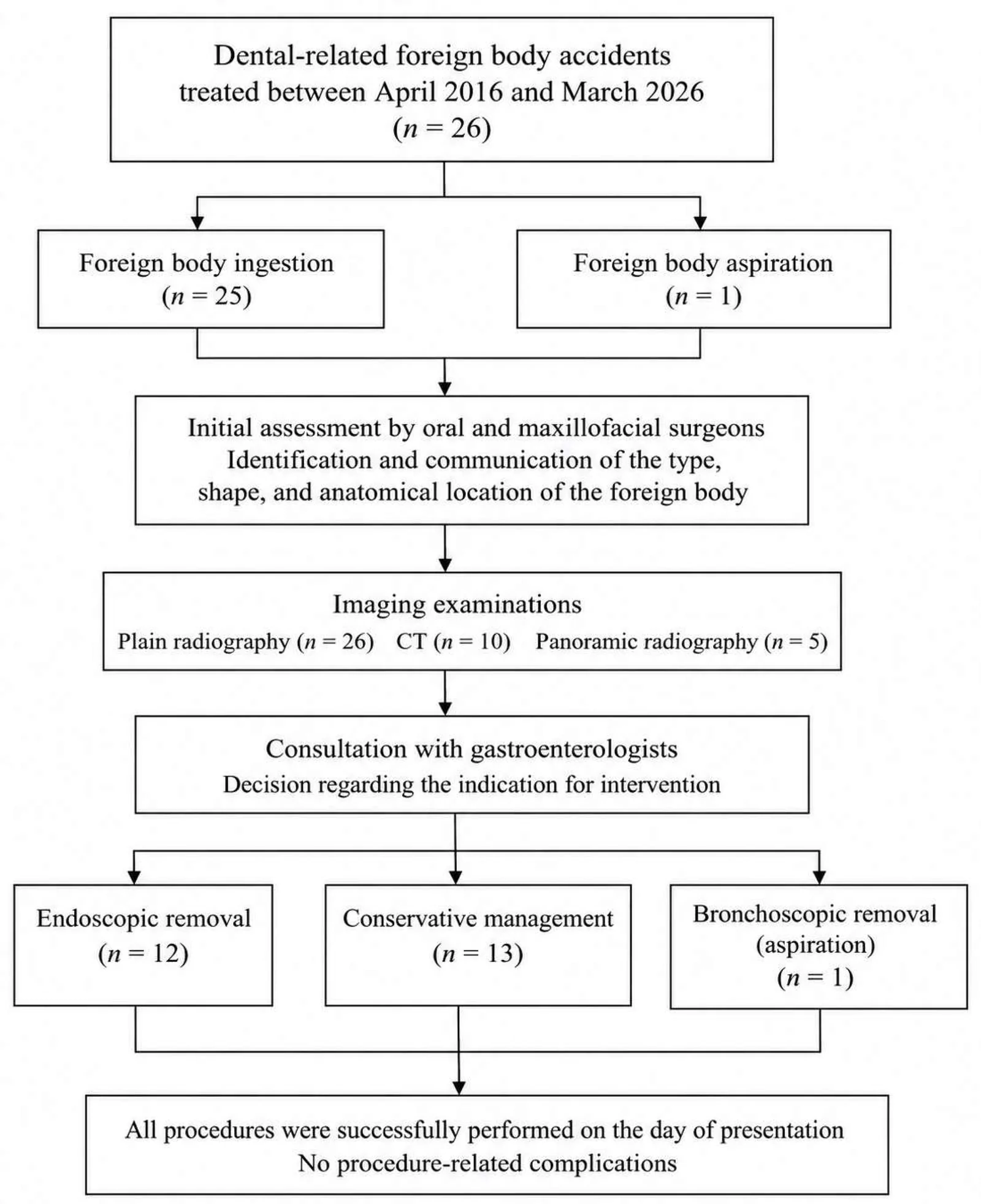
The clinical management pathway for patients with dental-related foreign body accidents. The patients underwent an initial assessment, imaging examinations, multidisciplinary consultation, and appropriate management according to the clinical situation. No procedure-related complications were observed.

**Table 1:** The characteristics of the 26 patients with dental-related foreign body accidents.

Variable	Overall (*n* = 26)
Age, yrs, mean ± SD	58.3 ± 26.6
Age group, yrs; *n* (%)	
<18	3 (11.5)
18–64	12 (46.2)
≥65	11 (42.3)
Sex, n (%)	
Male	16 (61.5)
Female	10 (38.5)
BMI, kg/m^2^, mean ± SD	22.0 ± 4.0
Accident circumstances, n (%):	
During dental treatment	19 (73.1)
Outside dental treatment	7 (26.9)
Referral route, n (%):	
Dental clinic	12 (46.2%)
Emergency transportation	2 (7.7)

**Table 2:** Characteristics of the foreign bodies (n = 26).

Variable	Overall (*n* = 26)
Foreign body category	n (%)
Restorative materials	15 (57.7)
Prosthetic appliances	3 (11.5)
Dental instruments	4 (15.4)
Orthodontic appliances	1 (3.8)
Tooth fragments	2 (7.7)
Others	1 (3.8)
Foreign body size	mean ± standard deviation (mm)
Long axis	16.8 ± 9.7
Short axis	7.3 ± 3.8

**Table 3:** The clinical management and outcomes of the 26 patients with foreign body accidents.

Variable	*n* (%)
Plain radiography *	26 (100.0)
Panoramic radiography	5 (19.2)
Computed tomography	10 (38.5)
Gastroenterology evaluation	17 (65.4)
Endoscopic removal	12 (46.2)
Bronchoscopic removal	1 (3.8)
Follow-up abdominal radiography	11 (42.3)
Hospitalization	3 (11.5)
Complications	0

* Including radiographs obtained at referring institutions.

## Data Availability

The data presented in this study are not publicly available because they contain information that could compromise the privacy of the research participants. De-identified data may be available from the corresponding author upon reasonable request and with permission of the Institutional Review Board.

## Supplementary Materials

The following supporting information can be downloaded at https://www.mdpi.com/article/10.3390/healthcare14193164/s1, Table S1: Individual clinical characteristics, foreign body characteristics, management, and outcomes of patients with dental-related foreign body accidents.
